# Live Fast, Die Young: Life History Traits of an Apex Predator Exacerbate the Ecological Impact of a Toxic Invader

**DOI:** 10.1002/ece3.70625

**Published:** 2024-11-29

**Authors:** Georgia Ward‐Fear, Gregory P. Brown, Lachlan Pettit, Lee‐Ann Rollins, Richard Shine

**Affiliations:** ^1^ School of Natural Sciences Macquarie University Sydney New South Wales Australia; ^2^ School of Biology, Earth and Environmental Sciences University of New South Wales Sydney New South Wales Australia

**Keywords:** *Bufo marinus*, ecological impact, invasive species, pace of life, Varanidae

## Abstract

We studied a population of large varanid lizards (yellow‐spotted monitors *Varanus panoptes* ) on a floodplain in tropical Australia. Growth records from radio‐tracked lizards show that despite their large adult body sizes (to > 7 kg in males), these lizards attained sexual maturity at less than 1 year of age and rarely lived for more than 2 years (females) or 4 years (males), even before mortality increased due to the arrival of toxic cane toads ( *Rhinella marina* ). This is a “faster” life history than has been reported for other species of large monitors. Growth was especially rapid in males during the wet season. The low survivorship prior to toad invasion was due to predation by pythons; communal nesting by female varanids may render them especially vulnerable. The life history of yellow‐spotted monitors requires high feeding rates, favouring the evolution of “risky” tactics such as consuming novel prey items (such as cane toads); and the combination of high abundance (> 20 adult lizards per square kilometre) and high feeding rates (> 9.9 kg of prey per lizard per annum) means that these giant lizards play a critical role in energy and nutrient flow within the floodplain ecosystem. As a result, foodwebs with the yellow‐spotted monitor as an apex predator are more vulnerable to disruption by cane toads than is the case in other parts of the toad's invasive range, where the varanid species affected by toads have “slower” life histories.

## Graphical abstract

We show that large floodplain monitors reach sexual maturity surprisingly rapidly, and females rarely live for more than 2 years. This fast life history requires novel reproductive strategies and high feeding rates, favouring the evolution of “risky” tactics such as consuming novel prey items (such as invasive toxic cane toads). As a result, foodwebs with the yellow‐spotted monitor as an apex predator are more vulnerable to disruption by cane toads than is the case in other parts of the toad's invasive range, where the varanid species affected by toads have “slower” life histories.

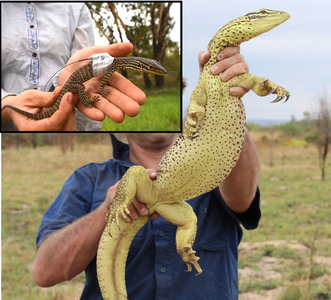

## 1 Introduction

Biological invasions are a major cause for biodiversity loss worldwide (e.g., Clavero and García‐Berthou 2005; Gallardo et al. 2019). Most scientific attention has been on the impacts of invaders on specific taxa (e.g., Fukuda et al. 2016; Potgieter et al. 2020), but the effects of invasions also cascade through foodwebs (e.g., O'Loughlin and Green 2017; Feit et al. 2018; Bryant, Beachy, and Boves 2020). Thus, for example, an invader's impact on a keystone predator or ecosystem engineer could have wider effects than would a similar numerical impact on other native species. Increasingly, invasion biology seeks to understand and rectify the nuanced impacts that invaders impose on recipient systems. Instead of focusing solely on the overall abundance or persistence of an impacted native species across their range, we also need to quantify the nature and magnitude of impacts at the population and community level; the loss of ecosystem functions; and the interplay between animal behaviour and vulnerability to threatening processes (Ricciardi et al. 2021; Haubrock et al. 2024). To understand variation in invader impacts, then, we need to consider attributes of the species that are affected, and how changes in abundance of those taxa affect other components of local ecosystems (e.g., Doody et al. 2006).

One trait that might influence the wider impact of an invasion is the life history of taxa that are directly affected. For example, the continuum between “fast” and “slow” demographic tactics is a critical dimension of interspecific variation, termed the “pace of life” (Réale et al. 2010). Individuals of a “fast” species grow rapidly, mature early, breed frequently, and rarely live for long, whereas individuals of a “slow” species grow less rapidly, delay maturation, breed infrequently, and survive to breed many times (e.g., Allen, Street, and Capellini 2017). Indeed, many invaders themselves possess fast life histories, enabling their rapid establishment and flexibility in novel environments (Capellini et al. 2015); but what remains unclear is how the life‐history traits of native species in recipient systems influence their vulnerability to those invaders (Ricciardi et al. 2021).

The “pace of life” theory correlates facets of life history, physiology, and behaviour (Réale et al. 2010) and has been demonstrated in many systems, both within and between species. The selective forces acting on fast life histories favour higher metabolic rates (Auer et al. 2018) and resource acquisition (Nakayama, Rapp, and Arlinghaus 2017). In turn, species can evolve behavioural characteristics such as higher rates of exploration (Rádai, Kiss, and Barta 2017) and taking risks whilst foraging (Sol et al. 2018) – for example, by exploiting more open habitats or by consuming novel (and thus, potentially dangerous) prey items. This constellation of traits associated with “fast” life histories might have two important ecological consequences: (i) the higher feeding rate of a “fast” species may increase its impacts on prey taxa, and (ii) species with “fast” life histories may be at greater risk when a novel toxic prey species invades their habitat. In short, the wider ecological impact of an invasion may depend on the demographic traits of species that are directly affected by the invader.

The spread of cane toads ( *Rhinella marina* ) through tropical Australia provides an ideal study system with which to explore these ideas. Native predators that cannot tolerate the distinctive defensive chemicals of cane toads are killed if they attempt to ingest a toad, causing massive reductions in abundances of taxa such as varanid lizards, bluetongue skinks, freshwater crocodiles and northern quolls (Letnic, Webb, and Shine 2008; Price‐Rees, Brown, and Shine 2010; Shine 2010). Declines of > 90% in the numbers of formerly abundant varanid lizards may be especially significant, because these giant lizards play critical roles as predators, scavengers and ecosystem engineers in many Australian ecosystems (Doody et al. 2015, 2020; Pettit, Ward‐Fear, and Shine 2021). However, impacts are highly variable; for example, yellow‐spotted monitors ( *Varanus panoptes* ) across tropical Australia exhibit long‐sustained declines whereas lace monitors ( *V. varius* ) in eastern Australia exhibit minor and short‐term impacts (Pettit, Crowther, et al. 2021). At least part of that difference in impact might be due to life histories of the varanid species concerned, but detailed information on these traits is scarce. To explore these issues we measured life‐history traits of yellow‐spotted monitors at a floodplain in tropical Australia, both before and after the arrival of toxic cane toads. We then compared the extent of these impacts to those exhibited by other species that have been impacted by toads in Australia. In combination, our results clarify how an animal's “pace of life” can influence both its vulnerability to a toxic invasive species, and the wider ecosystem‐level impacts of population declines wrought by such an invader.

## 2 Materials and Methods

### 2.1 Field Site and Study Species

The Kimberley region of tropical Australia experiences a “wet‐dry” climate. Rainfall is concentrated in a brief (4‐month) “wet season” (700 of 835 mm total for Kununurra, Western Australia: Bureau of Meteorology 2023). The annual monsoonal rains create large open floodplains around rivers, fringed by savannah woodland that grades into spinifex grassland in higher drier sites (Payne and Schoknecht 2011). Air temperature is consistently high (mean air temperatures exceeds 35°C in 8 months per annum). We worked across one such floodplain (Oombulgurri: 16,000 ha, 15°08′34S, 127°52′36E), 39 km northwest of Wyndham, where cane toads arrived towards the end of the wet season in April 2014 (see Ward‐Fear et al. 2016 for details). Because the toads caused high mortality in yellow‐spotted monitors, our estimates of lizard abundance and of ages at mortality in the current paper are based only on data from the first year (before toads arrived). Growth rates and ages at maturity of the monitors were apparently unaffected by toads (based on preliminary analyses), so we use the entire dataset for these variables. Our conclusions are qualitatively unaffected by inclusion versus exclusion of data from the second year.

**FIGURE 1:**
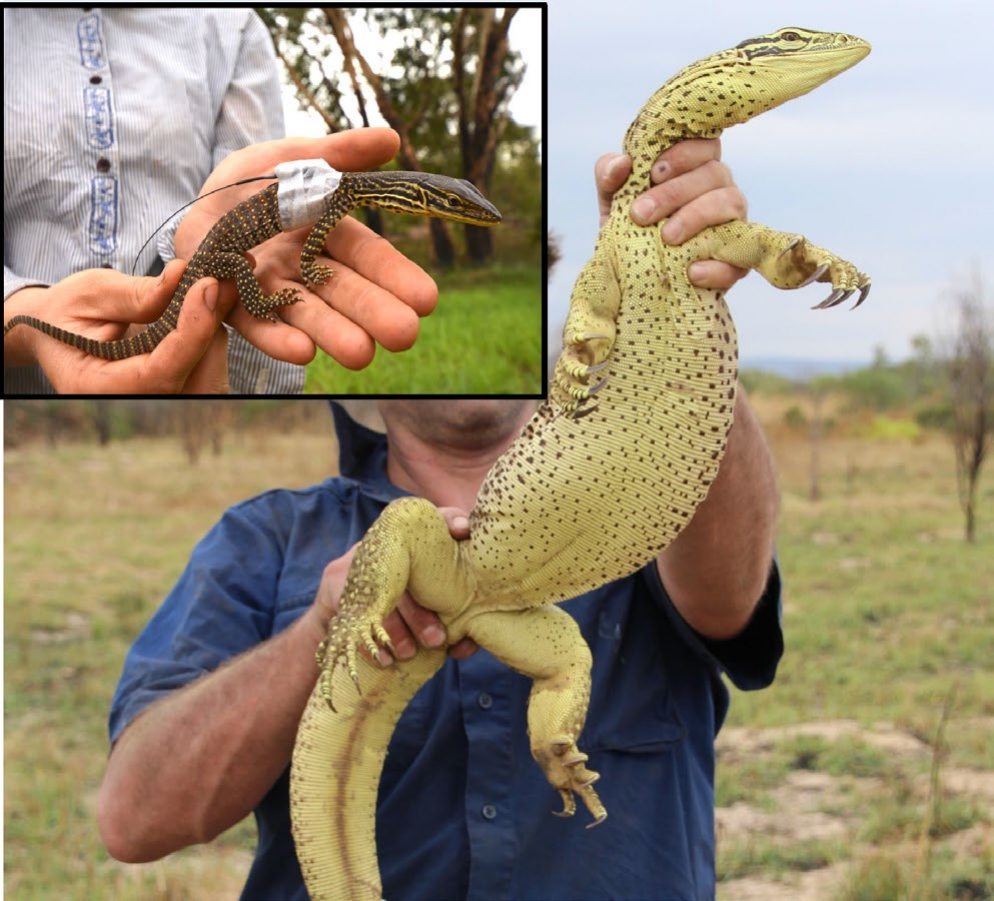
A male yellow‐spotted monitor *Varanus panoptes* that was first captured as a hatchling in November 2013 (inset), and radio‐tracked as it grew into a large adult over 18 months (pictured later, in May 2015). Photo credit: Georgia Ward‐Fear.

The yellow‐spotted monitor (“goanna”) is tropical Australia's largest lizard species, with males attaining snout‐vent‐lengths (SVLs) of > 600 mm, and body masses of > 7 kg (Pianka and King 2004; see Figure 1). Adult males grow > 3 times heavier than females (Shine 1986). Hatchlings average 140 mm SVL (Pianka and King 2004), and maturation is attained at around 390 mm SVL in males and 310 mm SVL in females (Shine 1986). Yellow‐spotted monitors are active for most of the year, but estivate in burrows during the late dry season (July–November, depending on local conditions: Christian et al. 1995), emerging with the first monsoonal rains. At our study site mating occurs in the mid‐wet season (December to February, but instances recorded in May), with oviposition in May/June and hatching in November to January (GWF, unpublished data). Female yellow‐spotted monitors lay eggs in multi‐chambered communal burrows that can be more than 4 m deep (Doody et al. 2014, 2020). Clutch sizes range from 6 to 14 eggs (Pianka and King 2004); we recorded one clutch of 9 eggs in a female that we dissected after she was consumed then regurgitated by a black‐headed python ( *Aspidites melanocephalus* ) in April. Yellow‐spotted monitors are generalist predators and have been identified as a keystone species, regulating trophic interactions in floodplain foodwebs (Shine 2010; Brown et al. 2013; Doody et al. 2015; Ward‐Fear et al. 2016).

### 2.2 Radiotelemetry

Between November 2013 and January 2016 we radio‐tracked 110 yellow‐spotted monitors (Female lizards *n* = 52; Male lizards *n* = 58). During 15 three‐week‐long field trips, we searched for monitors between 05:00 h and 11:00 h each day; hence, new individuals were recruited to the study through time. By collaborating with indigenous rangers, we were able to collect lizards exhibiting a wide array of behavioural phenotypes (Ward‐Fear et al. 2018, 2019).

Monitors were captured by hand and transported back to the research station where we recorded SVL and body mass and took tissue samples from the tail tip for genetic sex determination (see Appendix S1 for methodology). We attached a Very High Frequency (VHF) radio transmitter to the tail of each monitor (Holohil RI‐2B, 15 g, < 5% total body mass) following the methods of Madsen and Ujvari (2009) and released the lizard back into the field at its point of capture within 6 h; telemetry began 3 days post‐release. We tracked monitors at least twice per field trip (but also opportunistically) for as long as they were alive and could be located (mean number of observations per individual 12.2, range 5–40 observations). Throughout the study we opportunistically recorded information on behaviour, ecology and life‐history attributes of the animals.

### 2.3 Abundance

We estimated minimum population sizes in the first year of our study (before cane toads arrived) using a combination of lizards captured for telemetry, plus other individuals seen but not captured at the same sites. For the purposes of abundance calculations, we divided the wider floodplain into four segments, each associated with a separate riparian system. Searches for goannas occurred along rivers, where we counted every unmarked individual seen during morning surveys. To avoid counting uncaptured individuals more than once each day, we conservatively scored an animal as “already sighted” if it was similar in size and location to animals previously seen. Our index of minimum abundance per site was the number of yellow‐spotted monitors known to be living in an area at any one time. In addition to the 85 animals that we captured in year one, we made an additional 137 sightings of unmarked individuals. We encountered an average of one unmarked adult monitor per 2 h of search effort, even after most of our known individuals had been caught and were being tracked. Based on weekly encounter rates of unmarked individuals at each site we added an additional 5–10 adult animals in total, to our local count data. This conservative “correction” was designed to compensate for the high numbers of unmarked individuals.

We mapped surface area polygons of the search areas around each riparian system and calculated the minimum density of yellow‐spotted monitors per square kilometre in each of the four sites. We mapped the surface area of wet‐seasonal watersheds based on vegetation imagery, ground truthing and locations of radio‐tracked goannas across the floodplain. Based on these data we calculated the minimum density of adult monitor lizards per square kilometre in each of four sites (subsections of the floodplain).

### 2.4 Calculation of Growth Rates and Ages

We calculated growth rates using repeated measurements of SVL and body mass (g) taken on 17 males, 11 females and 4 hatchlings (total *N* = 32). Larger individuals were measured every three to six months, whereas juveniles were measured as often as possible. From the SVL measurements we calculated a von Bertalanffy (1957) curve to estimate growth rates (size vs. age); see Appendix S1.

### 2.5 Reproduction and Mortality

**FIGURE 2:**
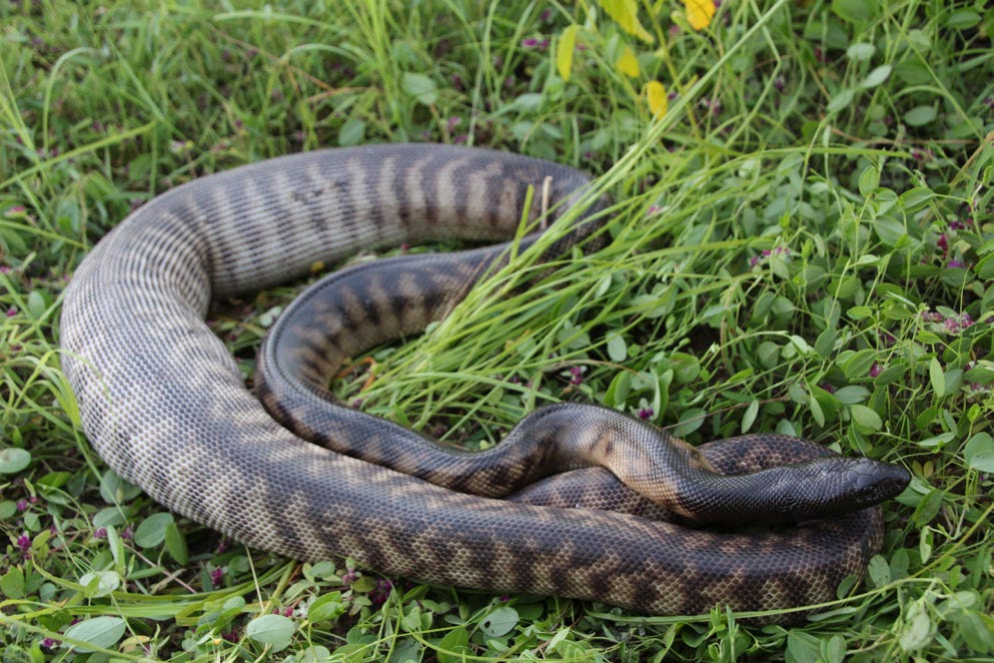
One of our radio‐tracked yellow‐spotted monitors ( *Varanus panoptes* ) was consumed by this black‐headed python ( *Aspidites melanocephalus* ). We tracked this python until it passed the transmitter. Photo credit: Georgia Ward‐Fear.

Over the course of the study we made 1172 observations of radio‐tracked monitors, only 7 of which were opportunistic encounters (i.e., a telemetered lizard was rarely sighted except by following the signal from its transmitter) plus 256 sightings of non‐telemetered animals. We inferred sexual maturity for lizards seen mating or engaged in courtship, or seen at nesting warrens (GWF, unpublished data). Gravid females were readily identifiable by their distended abdomens. We witnessed 27 instances of radio‐telemetered individuals with mates, one full sequence of courtship then mating (lasting at least 4 days), 13 instances of females nesting and an additional 18 instances of individuals thought to be engaging in mating and/or nesting in “warren” burrow systems. We also recorded 25 predation events on yellow‐spotted monitors by pythons (see Figure 2).

### 2.6 Energy Requirements and Rates of Prey Consumption

To estimate the annual rate of prey consumption by varanid lizards at Oombulgurri, we created energy budgets both on a per‐individual basis and also for the annual offtake of prey by yellow‐spotted monitors at our study site. Overall energy expenditure by an individual goanna during its annual period of activity (kJ per kg of goanna per day/month) was estimated as the sum of:

1. metabolic expenditure, from data on body mass (current study) combined with previous studies on mass‐specific metabolic rates of active and inactive *V. panoptes* in a climatically similar site (Christian et al. 1995);
2. allocation of energy to biomass growth, from growth rates in mass (calculated in the current study) combined with data on the energetic content of lizard tissue (Peterson et al. 1999); and
3. (for females only) allocation of energy to a clutch of eggs, based on mean clutch sizes and egg masses (from 6–14 eggs at 30‐80 g each) and caloric content of eggs (5.8 cal/mg–7.2 cal/mg Tinkle and Hadley 1975; Angilletta and Sears 2001).

To simplify our calculations, we averaged energy expenditure across the entire annual period of activity (November–July). For each sex, we created a monthly mean value by averaging the data for all our male and female animals. To calculate total intake of prey per lizard, we used published estimates of gross conversion efficiency for omnivorous lizards of 80% (the percentage of ingested energy that is available for allocation to metabolism, growth, and reproduction: Buffenstein and Louw 1982; Wehrle and German 2023) and caloric content relative to mass for the most common prey species consumed by *V. panoptes* at our study site (see Ward‐Fear, Shine, and Brown 2020 for dietary data, Grayson et al. 2005 for caloric content relative to mass). Because *Varanus panoptes* is a generalist predator, consuming multiple types of prey each day, we averaged the values for all prey items to create a standardised value of “gm of mixed prey” required to support the energy budgets of male and female lizards. To calculate total offtake of prey per square kilometre, we multiplied the per‐capita consumption by the estimated upper and lower abundance of yellow‐spotted monitors on the Oombulgurri floodplain.

### 2.7 Comparison With Sympatric Lace Monitor ( *Varanus varius* )

We directly compared the life‐history and dietary traits of *Varanus panoptes* with those of a morphologically similar species, the lace monitor ( *Varanus varius* ) in eastern tropical Australia (a region where the two large varanid taxa are broadly sympatric). We then looked at the magnitude of cane toad‐induced impact on both species over the long term.

### 2.8 Statistical Analysis

Normality and homogeneity of variance were confirmed for all variables. All continuous variables (age, number of days tracked, SVL, mass etc.) were transformed with natural log (Ln) prior to analyses.

#### 2.8.1 Seasonal Growth Rates

We ran full factorial ANOVAs with growth rates (i.e., the increase in mass between successive captures, standardised as gm/day) as our dependent variable, and independent variables of sex, season (wet = November to April; dry = May to October) and the interaction between the two.

#### 2.8.2 Reproductive Age and Survivorship

we analysed data on the age of all telemetered individuals that were recorded as mating or nesting during the study. The dependent variable in our ANOVA was the inferred age of the animal at the time of mating (obtained from the von Bertalanffy growth curve at initial capture and adding number of days tracked to that point) and the independent variable was sex (male/female). To explore sex differences in survivorship, we conducted ANOVAs to compare the mean number of days animals were tracked alive and the inferred ages of those animal at their time of death.

We then created a population profile by calculating probable age at first capture from body size (using the von Bertalanffy growth curve then adding the number of days that the animal was tracked until it died). We used ANOVA to compare the average “ages” of males and females within the population, as well as the distribution of age classes between the sexes.

## 3 Results

### 3.1 Rates of Growth

**FIGURE 3:**
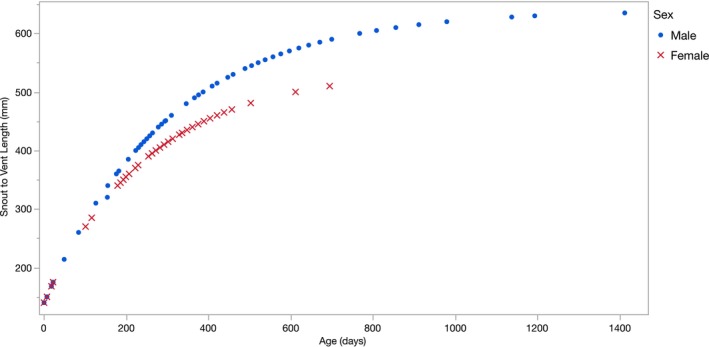
A von Bertalanffy growth curve calculated from rates of increase in snout‐vent length of 32 radio‐tracked yellow‐spotted monitors, *Varanus panoptes* (M: 17, F: 11, J: 4). Males and females begin to diverge in growth rates at around 160 days of age (5.3 months), when they are about 300 mm SVL. Curves were not extrapolated past the highest SVL measured for each sex in our population.

Yellow‐spotted monitors grew rapidly with growth rates diverging between the sexes at around 300 mm SVL, when animals were about 160 days old (5.3 months; see Figure 3 and Figure S1). Based on their emergence from natal nests in the early wet season, this sex‐based divergence in rates of growth would occur near the end of the animal's first wet season (March–May). During the wet season, males increased in mass faster than did females (mean 7.4 vs. 3.3 g/day; *F*_1,31_ = 4.59, *p* = 0.04), and males grew faster in the wet season than in the dry season (mean 7.4 vs. 2.2 g/day; *F*_1,29_ = 4.37, *p* = 0.046). We do not have data on dry‐season growth rates for females (see Figure 1).

Noteworthy records of rapid mass increase include an adult male gaining 2.36 kg over 99 days mid‐wet season (increasing his body mass by 145%, from 1.63 kg to 3.99 kg), and a young male gaining 1.28 kg over 150 days in the wet season (increasing his body mass by 312%, from 410 g to 1.69 kg; see Table S1). Fitting these data to a von Bertalanffy curve, we estimate that both sexes attain reproductive size (310 mm females, 390 mm males: Shine 1986) at around 200 days of age (< 7 months; Figure 3).

### 3.2 Inferred Ages of Reproducing Lizards

We recorded 13 females reproducing within 1 year of birth (i.e., in the wet season following the one in which they were hatched); another nesting female was 2 years old. Of 13 males that we recorded mating, only two were within 1 year of birth; the remaining 11 were 2 years or older (Mean age F 403 days, M 729 days; min F 203 days, M 277 days; Max F 862 days, M 1153 days; sexes differ in mean age at mating: *F*_1,26_ = 12.94, *p* = 0.001). See Table S2 for summary of morphology and age of sampled lizards.

**FIGURE 4:**
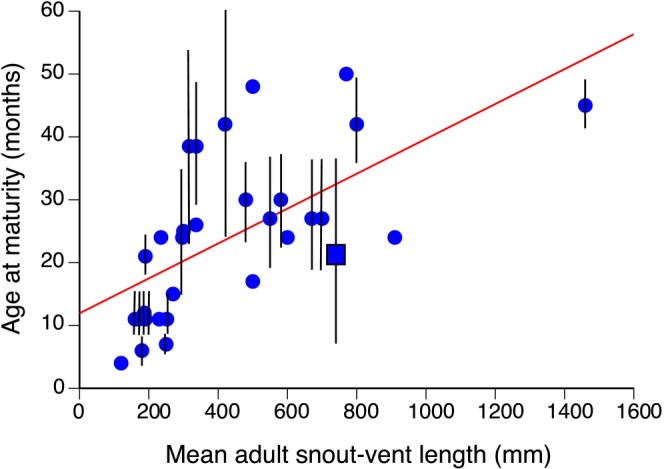
Relationship between mean adult body size and age at maturation in 31 species of varanid lizards, based on data from the review by Auliya and Koch (2020). The graph shows age at maturity (primarily records from captive animals) plotted against mean body size (snout‐vent length, = SVL) per species. Mean body sizes are average of data for males and females, where available, and vertical lines show the range of ages at maturation recorded for each taxon. Dots show mean values, and the square and associated vertical line shows data for yellow‐spotted monitors *Varanus panoptes* .

A review of published data suggests that yellow‐spotted monitors are unusual among large‐bodied varanid lizards in maturing and reproducing at such a young age. Most data for monitors are based on captive lizards, which tend to reproduce at younger ages than do conspecifics in the wild (based on species for which data of both types are available: Auliya and Koch 2020). Nonetheless, most large‐bodied varanids delay reproduction until they are 18–24 months of age (Figure 4). Yellow‐spotted monitors (shown by the square symbol in Figure 4) are an exception in this respect, with one record of reproduction in captive animals at 6.5 months of age (Nabors 1997; see square symbol in Figure 4), the same age as we have inferred for first reproduction in the wild. Other reports, however, suggest longer delays before maturation (Auliya and Koch 2020; Figure 4).

### 3.3 Survivorship

The interval between capture and death averaged shorter for female lizards than for males during our radio‐tracking study (mean days tracked alive: F 108 days; M 200 days; *F*_1,51_ = 4.56, *p* = 0.038).

Prior to the arrival of cane toads, all 14 deaths that we recorded of adult radio‐tracked yellow‐spotted monitors were due to predation by black‐headed pythons ( *Aspidites melanocephalus* , *N* = 5) or olive pythons (*Lialis olivaceus*, *N* = 2) with a further seven deaths due to unknown python species (i.e., transmitters were found in python faeces rather than inside a python). On average, female monitors killed by pythons were younger than were the males (Mean F 433 days; M 735 days; *F*_1,14_ = 13.5, *p* = 0.003) and all seven females were close to warren burrows when killed (and hence, likely were nesting). Another nine telemetered monitors died due to python predation later in this study, but we excluded these animals from the above analyses because toad‐induced mortality modified sex and size distributions within the monitor population (Ward‐Fear, Brown, and Shine 2024).

### 3.4 Age Profile of the Population

**FIGURE 5:**
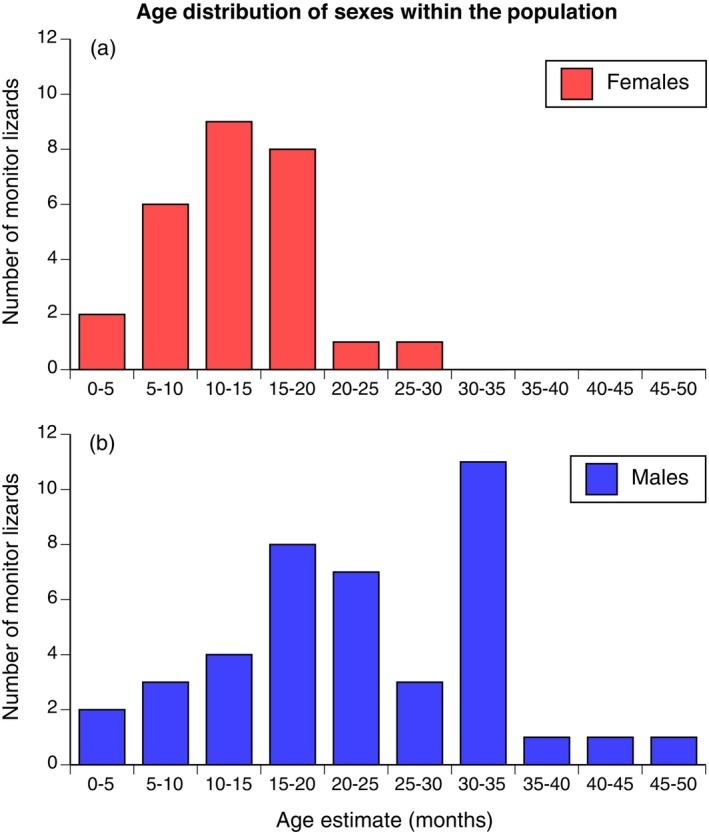
The frequency distribution of age groups (as inferred from body sizes and growth rates) of yellow‐spotted monitors. To obtain this age estimate we calculated probable age at first capture from body size (using the von Bertalanffy growth curve, Figure 3) then added the number of days that the animal was tracked until it died.

Mean ages were greater for males than for females (Mean F 263 days; M 465 days; *F*_1,112_ = 22.97, *p* < 0.0001) and males had a wider spread in age classes (Figure 5). Most females were < 18 months old, with only four individuals in older classes. In contrast, several males were 3 years old, and one was estimated to be 4 years old when it died (Figure 5).

### 3.5 Abundance

Radiotelemetry showed that yellow‐spotted monitors had strong site fidelity (Ward‐Fear, Shine, and Brown 2020). The minimum number of adult yellow‐spotted monitors known to be present in each river system at the same time was as follows: Dorrimon 15 monitors per sq. km; Allison River 20 per sq. km; Larramon 22 per sq. km; Patrick River 33 per sq. km. From our watershed polygons (i.e., ground‐truthed areas of complex and resource‐rich habitat favoured by these monitors) we calculated that the Oombulgurri floodplain contains 76.4 km^2^ of high‐quality habitat. Based on the lowest and highest density records of adult yellow‐spotted monitors present concurrently at sites, we estimate that the floodplain supported between 1146 and 2521 adult yellow‐spotted monitors at any one time during our study.

### 3.6 Energy Requirements and Rates of Prey Consumption

To sustain maintenance, growth and reproduction, female lizards would need an average FMR of 183.04 kJ/day (range 38.12–310.73 [1.4 kg lizard]; see Appendix S1). Females would need to increase their metabolism by 45%–52% to produce an annual clutch of eggs. For male lizards, we estimated an average FMR of 272.3 kJ/day (range: 66.87–503.82 [3.9 kg lizard]). The largest and fastest‐growing males had nearly double the energy requirements (approx. 500 kJ/day).

To support these energy expenditures, an average‐sized adult female monitor (1.08 kg) would need to consume 37 g of mixed prey per day, and a male (2.14 kg) would need to consume 55 g per day. A large male monitor (> 3.5 kg) would need 100 g of prey per day. Across the nine‐month annual activity period of these monitors at our field site, an average‐sized female monitor would require 9.9 kg of prey per annum, an average‐sized male would require 14.8 kg and a large male would require 27 kg (see Table 1). Based on the mass and caloric content of prey species, the daily prey intake of an average‐sized female yellow‐spotted monitor would translate into approximately 17 spiders, 7 beetles, 2 frogs, 1 lizard or 0.25 rats, an average‐sized male would eat approximately 25 spiders, 10 beetles, 3 frogs, 1.3 lizard or 0.35 rats, and a large male would require approximately 46 spiders, 19 beetles, 5 frogs, 2.4 lizards or 1 rat (see Table S3 for monthly totals per individual lizard).

**TABLE 1:** Estimates of biomass consumption by the population of yellow‐spotted monitors ( *Varanus panoptes* ) at our field site, the Oombulgurri floodplain. We calculated and averaged monthly energy budgets for average‐sized males and females (and for larger males) based on growth rates measured in our study animals. Using site‐specific dietary information for this generalist predator, we calculated how many grams of “mixed prey” would be required to support an average male, female and large male lizard over three timescales: per day, per month, and throughout their annual activity period (provided in kilograms, shown in bold). We then modelled the collective biomass consumption of the adult goanna population each year (in tonnes, shown in bold), at the lower (1146 animals) and upper (2521 animals) population estimates.

Calculation	Time period	Female (5491.4 kJ/month)	Male (8170.2 kJ/month)	Larger male (15,100 kJ/month)
Biomass of mixed prey required	Per day	37 g	55 g	100 g
	Per month	1098 g	1634 g	3019 g
	Per year	9991 g	14,865 g	27,473 g
		**9.99 kg**	**14.87 kg**	**27.47 kg**
Population of average adult lizards with 50/50 sex ratio	Annual activity period	Lower pop. estimate	**14.25 t**	Prey biomass offtake collectively
		Upper pop. estimate	**31.31 t**	

Multiplying these individual rates of annual prey consumption by population estimates (with a 50/50 sex ratio) for our field site, we calculate that prior to toad invasion the yellow‐spotted monitors at our study site consumed between 14.3 t of prey per annum (at the lower population estimate of 1146 adult lizards) and 31.3 t per annum (at the higher estimate of 2521 adult lizards). This population estimate does not include juvenile lizards. Rates of prey consumption are based on the caloric requirements of average‐sized males and females at our study site.

### 3.7 Comparison With Sympatric Lace Monitors ( *Varanus varius* )

Despite their similar sizes and diets, the two varanid species differ in multiple life‐history traits as well as in their resilience to cane toad invasion (Table 2). Toad invasion has resulted in a much greater population decline, and less recovery, in the species with a “faster” life history ( *V. panoptes* ) than in the congener with a “slower” life history ( *V. varius* ).

**TABLE 2:** Comparison of life history, behaviour and cane toad impact between the yellow‐spotted monitor ( *Varanus panoptes* ) and the lace monitor ( *Varanus varius* ). Despite attaining similar sizes, and living sympatrically in northeastern Australia, yellow‐spotted monitors exhibit a faster pace of life than do lace monitors, and exhibit greater long‐term population impacts from invasive cane toads. SVL, snout‐vent length.

Species	Yellow‐spotted monitor ( *Varanus panoptes* )	Lace monitor ( *Varanus varius* )	References
Diet specialisation	Seasonal generalist	Seasonal generalist	Losos and Greene (1988); Ward‐Fear, Shine and Brown (2020)
Maximum size	650 mm (SVL); 8 kg (avg 5 kg)	760 mm (SVL); 14 kg (avg 8 kg)	Thompson, Pianka, and JMcEachran (2001)
Age at sexual maturity	< 1 year	4–8 years	Ward‐Fear, Shine, and Brown (2024); Carter (1992)
Mean clutch size (wild)	11	8	Thompson, Pianka, and JMcEachran (2001)
Average longevity (wild)	3–8 years	> 20 years	This paper; Auliya and Koch (2020)
Field metabolism CO(2) g(−1) h(−1)	0.24 (early wet season)	0.05 (spring)	Christian et al. (1995), Guarino et al. (2002)
Pace of Life	Fast	Slow	This paper
Habitat use	Open	Wooded	Lei and Booth (2018)
Impact of cane toads	Major impact, no recovery	Low‐medium impact, rapid recovery	Jolly, Shine, and Greenlees (2016), Pettit, Crowther, et al. (2021)

## 4 Discussion

Intuition suggests that a giant lizard is likely to be old (because it will take a long time to attain that size), and that large predators will occur at low population densities (because their energy needs are too great to sustain high numbers: Colinvaux 1979). Those predictions are met by many species, but not all. For example, most diverse phylogenetic lineages include species with life histories that are much “faster” than those of similar‐sized related taxa (e.g., Miles and Dunham 1992; Webb, Christian, and Fisher 2002; FitzGibbon 2015); and even large predators sometimes occur at high population densities (e.g., Madsen et al. 2006; Ajtić et al. 2013). In the case of the yellow‐spotted monitor, our data show that these large lizards grow rapidly, generally die early, and can occur at high population densities. In turn, those attributes may exacerbate the assemblage‐level ecological impacts of invasive cane toads.

Compared to other large‐bodied species of lizards, yellow‐spotted monitors appear to be unusual in their “fast” growth and maturation. Of the few studies that have quantified rates of maturation in free‐ranging varanids, most have concluded that individuals of large‐bodied taxa delay maturation until they are at least 2 years old (e.g., *Varanus bengalensis* [> 3 years]: Auffenberg 1994; *V. komodoensis* [8–11 years]: Laver et al. 2012; *V. olivaceous* [2–3 years]: Auffenberg 1988; *V. niloticus* [> 2 years]: de Buffrénil and Hemery 2002; *V. salvator* [> 2 years]: Andrews 1995; *V. varius* [4–8 years]: Carter 1992). Only small‐bodied varanid taxa mature less than a year after hatching (as we have recorded for yellow‐spotted monitors), even in well‐fed captives under conditions that accelerate growth and maturation (see data in Auliya and Koch 2020; and see Figure 4). This correlation between life‐history pace and body size conforms to general patterns within the pace of life continuum (Dobson and Oli 2008). We note, however, that age at maturity may vary among populations within a species. Although Nabors (1997) reported maturation at around 6 months in yellow‐spotted monitors (Nabors 1997), as in our field populations, other sources have reported maturation at two to three years of age (Auliya and Koch 2020). Inter‐population variation in age to maturation and longevity is widespread in lizards (e.g., Wapstra, Swain, and O'Reilly 2001), including varanids (de Buffrénil and Castanet 2000; de Buffrénil and Hémery 2002). Female varanids tend to have shorter lifespans than conspecific males even in captivity, hinting at high costs of egg production and oviposition (Frýdlová et al. 2013). More generally, individuals of most lizard taxa appear to mature at one to two years of age, with age at maturity increasing with adult body size (e.g., Dunham and Miles 1985; Miles and Dunham 1992). Hence, the combination of large body size and rapid maturation in yellow‐spotted monitors is unusual.

Why do yellow‐spotted monitors have such “fast” life histories? The answer likely involves both phenotypic plasticity and adaptation. Age at maturation is highly plastic in reptiles, such that increased temperature and food supply accelerate growth and hence, maturity (e.g., Sorci, Clobert, and Belichon 1996). Riparian habitats on the Oombulgurri floodplain offer diverse and abundant prey (Ward‐Fear, Shine, and Brown 2020) and a consistently hot climate (Bureau of Meteorology 2023). Those conditions increase growth rates and thus decrease the time needed to achieve adult body size. In keeping with that proximate effect, many tropical reptiles exhibit faster growth and earlier maturation than do related taxa from cooler regions (e.g., *Oxyuranus* Shine and Covacevich 1983; *Malayopython* Shine, Harlow, and Keogh 1998; *Chlamydosaurus* Griffiths 1999; *Acanthophis* Webb, Christian, and Fisher 2002; *Tropidonophis* Brown and Shine 2002).

Local thermal conditions also can affect ages at maturation via selection (Adolph and Porter 1996). Life‐history theory predicts that stochastic variation in resource availability may increase the fitness advantages to early maturation (Engen and Sæther 2016); and that early maturation enhances individual fitness (lifetime reproductive output) under conditions that facilitate rapid growth but impose high levels of extrinsic mortality (e.g., Hutchings 1993). The Oombulgurri population of yellow‐spotted monitors satisfies these conditions. First, annual variation in the timing and amount of wet‐seasonal rainfall creates stochasticity of resource levels (e.g., Madsen et al. 2006). Second, rapid growth is possible because food is abundant and temperatures are high. Third, extrinsic mortality is frequent, with many of our radio‐tracked lizards (up to 650 mm SVL) being killed and consumed by pythons. The vulnerability of these giant lizards is surprising, but their burrows provide little opportunity for escape from an incoming python. Communal nesting in deep burrows may exacerbate that vulnerability, because the location of these nest‐sites is consistent across years (Doody et al. 2020; GWF, unpublished data) and hence is predictable. It would be interesting to study longevity of *V. panoptes* in other areas. For example, in sites where predatory pythons are less abundant, we expect that some individual monitors will live much longer than was the case at our Oombulgurri site.

High mortality rates mean that many female yellow‐spotted monitors reproduce in only a single season (Figure 5). That situation may help to explain the remarkable nesting ecology of this species, whereby females dig very deep (to > 4 m) burrows that provide stable moisture levels (Doody et al. 2020). Because adult female yellow‐spotted monitors are unlikely to survive to another nesting season, selection to maximise survival rates of embryos likely is intense: a female's fitness depends upon the offspring from her first clutch. The optimal life‐history tactic for a reproducing female with high exposure to extrinsic mortality (predation) may be to increase reproductive effort into activities such as nest‐digging, even to the point of compromising her own survival after laying (Frýdlová et al. 2013).

Many male monitors in our study population lived longer than females (Figure 5), and grew to substantially larger sizes. Continued growth in males may reflect a sexually‐selected advantage to large body size. Our data confirm that larger, older males obtained more matings than did their smaller, younger rivals (see Section 3). Consistent use of the same nesting sites may increase access to females for males as well as for predatory pythons, intensifying male–male rivalry (Pianka and King 2004). Thus, rapid maturation may cascade through to affect other aspects of the species' life history, ranging from deep‐nesting to extreme sexual size dimorphism.

The “fast” life history of yellow‐spotted monitors also may increase their vulnerability to cane toad invasion, because a large lizard must feed frequently in order to sustain high costs of metabolism, growth and reproduction. Such high feeding rates of generalist predators, in turn, favour risky foraging tactics, consistent with our comparison between yellow‐spotted monitors and lace monitors ( *Varanus varius* ) in tropical Australia. Although they are similar in mean body size to *V. panoptes* , lace monitors exhibit a slower pace of life. For example, they have a lower metabolic rate (Christian et al. 1995), grow more slowly and reach sexual maturity later (Carter 1992), and generally live longer in the wild (Auliya and Koch 2020). Those “slow” traits likely reduce the intensity of selection for early resource acquisition that promotes risky foraging tactics. Another key (and possibly causal) difference between the two large varanid species may lie in their preferred habitats. Yellow‐spotted monitors are found primarily in open habitats (Shine 1986; Ward‐Fear et al. 2018), where active searching for prey during daylight hours exposes animals to predators (Biro et al. 2004); and even their nocturnal burrows may be relatively easy for predators to locate in the open environments utilised by these large lizards. In previous work, we have shown that bolder individual *V. panoptes* remain closer to resource‐rich riparian zones and (perhaps as a result) experience higher rates of wet season predation (Ward‐Fear et al. 2018). Like yellow‐spotted monitors, lace monitors are large diurnal animals with extensive home ranges, but they predominantly inhabit forested habitats (Lei and Booth 2018) and climb trees when disturbed. Within populations, individual lace monitors that spend time in more open habitats are bolder (Pettit, Brown, et al. 2021). These patterns support the idea that by increasing exposure to predators, open habitats favour distinctive behavioural and life‐history traits in varanid lizards.

Risky foraging tactics may include a preparedness to consume novel prey items. Both yellow‐spotted and lace monitors are generalist species, consuming a similar diversity of prey types (including invertebrates, reptiles, frogs, mammals, eggs, birds, and carrion: Shine 1986; Losos and Greene 1988; Ward‐Fear, Shine, and Brown 2020). At the leading edge of the cane toad invasion, both varanid species have been reported to consume cane toads and to die as a result (e.g., Jolly, Shine, and Greenlees 2016; Ward‐Fear et al. 2016). Nonetheless, long‐term impacts of toad invasion differ between the two varanid species. Yellow‐spotted monitors have been virtually extirpated, whereas lace monitors remain abundant (Pettit, Crowther, et al. 2021). The resilience of lace monitors appears to arise from cautious foraging, whereby novel prey items (such as cane toads) are evaluated before being swallowed (Jolly, Shine, and Greenlees 2016). Bolder individuals of both species are disproportionally at risk from cane toads (at least initially; GWF ref., Pettit, Brown, et al. 2021). Collectively, these comparisons show that the yellow‐spotted monitor is faster lived, less risk‐averse and more heavily impacted by toad invasion than is the lace monitor.

The two large varanid species also differ in the degree to which their extirpation affects sympatric fauna. First, unlike the lace monitor, the yellow‐spotted monitor is an “ecosystem engineer”; it digs extensive burrow systems for nesting (Doody et al. 2014) and frequently excavates shorter burrows for nocturnal refuges (Ward‐Fear et al. 2018). Those burrows offer distinctive abiotic conditions and are exploited by many other species. Second, the high feeding rates, generalised diet and high abundance of yellow‐spotted monitors mean that population collapse of this species (due to cane toad invasion) ramifies through the food web, inducing cascading effects at lower trophic levels (Shine 2010; Brown et al. 2013; Doody et al. 2015; Ward‐Fear et al. 2016; Ward‐Fear, Shine, and Brown 2020). Increased survival rates of species consumed by monitors modify the structure and dynamics of the floodplain faunal assemblage (Ward‐Fear, Shine, and Brown 2020). Lower abundances and feeding rates of lace monitors mean that the ecosystem‐wide effects of their removal (due to poisoning by toads) may be less than for yellow‐spotted monitors (but see Pettit, Ward‐Fear, and Shine 2021).

Another varanid species for which a comparison is possible is a species in a cooler climate. Annual food intake by *Varanus rosenbergi* on Kangaroo island in South Australia has been estimated at around 4.7 kg per annum for a 1 kg lizard (Green, Dryden, and Dryden 1991), about half that consumed by an average adult female yellow‐spotted monitor (~1 kg at our site also). If we compare more widely, looking at endothermic as well as ectothermic predators, two other taxa with similar generalist diets that occur in Oombulgurri are the dingo (*Canis lupis*) and the black kite ( *Milvus migrans* ). An adult dingo requires around 302,400 kJ/month (Tatler et al. 2021) and a black kite requires 12,339 kJ/month (Tarboton 1978). Although energy intake per individual is higher for these endotherms than for monitors (median 8170 kJ/month), population densities are far lower for dingos (0.15 individuals/sq. km: Gabriele‐Rivet et al. 2020) and black kites (0.2/sq. km: Gosper and Holmes 2008) than for yellow‐spotted monitors (median = 25 monitors/sq. km, from our data). Accordingly, the total energy offtake by yellow‐spotted monitors is much higher than for the endotherms. To support their monthly energy requirements, a population of dingos would consume 50 pale field rats ( *Rattus tunneyi* ) per square kilometre, a population of black kites would consume three rats, and a population of yellow‐spotted monitors would consume 258 rats.

The link between “fast” life histories, vulnerability to invasion, and wider impacts of invasion, is not unique to monitors. Another native predator decimated by cane toad invasion exhibits an even “faster” life history. Once abundant across the wet‐dry tropics (Oakwood 2008), populations of cat‐sized marsupial carnivores (Northern Quolls *Dasyurus hallucatus* ) have decreased dramatically due to toad invasion (e.g., O'Donnell, Webb, and Shine 2010; Indigo et al. 2023). Quolls of both sexes mature at a year of age, and rarely survive for more than 18 months (Oakwood 2000) and thus, like the monitors, they eat vast amounts of food, of a diverse array of prey types, and even a single year's recruitment failure is devastating for population viability (Moro, Dunlop, and Williams 2019). Hence, many of the arguments we have made about yellow‐spotted monitors apply with equal force to quolls. Other predators fatally poisoned by toads (such as freshwater crocodiles, *Crocodylus johnstoni* ) have “slower” life histories, potentially reducing both vulnerability to toads (due to lower feeding rates by predators) and the trophic cascades resulting from predator mortality (because mortality is concentrated on only a subset of crocodile size classes: Fukuda et al. 2016; Ward‐Fear et al. 2024).

More generally, a species' life history will influence how it experiences different conservation challenges (Albaladejo‐Robles, Böhm, and Newbold 2023). For example, species with faster life histories often adapt better to anthropogenic habitat degradation (which indirectly changes carrying capacity), but slower‐lived species may deal better with habitat modification than with excessive harvesting, which impacts survival directly (González‐Suárez, Gómez, and Revilla 2013). Furthermore, a species can alter its pace of life in response to environmental stressors (Prabh et al. 2023). “Invasions” are not as easy to categorise, because impacts from invasive species are diverse in nature, and depend upon the unique characteristics of the invaded ecosystems as well as of the invader itself. Overall, we know very little about how an animal's pace of life influences its vulnerability to invader impacts. The native taxa impacted by invasive species encompass an enormous diversity in life‐history traits, and it may often be the case that “faster” life histories:

1. increase vulnerability of a native species by favouring relatively unselective feeding at high rates (as in the current study) or via other pathways such as high activity levels (increasing exposure to invasive predators) or dependence upon high resource levels (which can be reduced by direct or indirect invader effects: Biro and Stamps 2008); and
2. magnify downstream impacts of the invader on foodwebs because a native species with a “fast” life‐history requires high rates of resource consumption to sustain rapid growth, early maturation and frequent reproduction (Auer et al. 2018). Such effects may ramify to higher as well as lower trophic levels, because invader impacts on a highly abundant organism or keystone species with high rates of energy throughput likely will have more effect on consumers of that organism than would be the case for slowly‐growing taxa at the same trophic level. These ideas suggest that including information on the life‐history traits of native species directly impacted by a biological invasion might help us to predict both the magnitude of direct impact, and the degree to which such an impact will cascade through foodwebs and hence, modify the broader biotic community.

## Author Contributions

**Georgia Ward‐Fear:** conceptualization (lead), data curation (lead), formal analysis (lead), investigation (lead), methodology (lead), project administration (lead), visualization (lead), writing – original draft (equal), writing – review and editing (equal). **Gregory P. Brown:** conceptualization (supporting), formal analysis (supporting), methodology (supporting), supervision (equal), writing – review and editing (supporting). **Lachlan Pettit:** conceptualization (supporting), data curation (supporting), formal analysis (supporting), methodology (supporting). **Lee‐Ann Rollins:** data curation (supporting), formal analysis (supporting), methodology (supporting), resources (supporting). **Richard Shine:** conceptualization (supporting), funding acquisition (lead), investigation (supporting), methodology (supporting), resources (equal), supervision (equal), writing – original draft (equal), writing – review and editing (equal).

## Conflicts of Interest

The authors declare no conflicts of interest.

## Supporting information


Appendix S1

## Data Availability

Data are publicly available via the DRYAD repository at https://doi.org/10.5061/dryad.jwstqjqks.
