# Supplementary material for: Live Fast, Die Young: Life History Traits of an Apex Predator Exacerbate the Ecological Impact of a Toxic Invader
Source: Ecol Evol. 2024 Nov 29;14(12):e70625. doi: 10.1002/ece3.70625 (PMC11606729; doi:10.1002/ece3.70625)
Supplement: Supplementary file 1 — Appendix S1 [file ECE3-14-e70625-s001.docx]

**Live fast, die young: life history traits of an apex predator exacerbate the ecological impact of a toxic invader**

**Georgia Ward-Fear^1C^, Gregory P. Brown^1^, Lachlan Pettit^1^, Lee-Ann Rollins^2^, Richard Shine^1^**

**Appendix**

**Materials and Methods**

### *Sex determination*

Morphological assessment of sex in varanids is unreliable, especially in small individuals (e.g., Auffenburg 1981). Genomic DNA was extracted using a Gentra PureGene Tissue Kit (Qiagen, Venlo, The Netherlands) following the manufacturer's instructions. Molecular sexing was conducted using primers *Ksex1* and *Ksex3,* originally developed for *Varanus komodoensis* (Halverson et al., 2002). Ten‐microliter PCRs included approximately 30 ng of DNA, 5 pM of each primer, 1× buffer, 2.5 mmol/L MgCl_2_, 300 μmol/L dNTPs, and 0.5 U AmpliTaq (Life Technologies, Carlsbad, California, USA). Cycling conditions included a 5‐min step at 94°C followed by 35 cycles of 94°C (45 s), 55°C (45 s), and 72°C (1 min), with a final extension step at 72°C for 7 min. Amplifications were visualized on a 2% agarose gel. Forty individuals of either known (from necropsy, N = 7) or inferred (from mating/nesting behaviour and size, N = 33) sex were used to verify the accuracy of molecular sexing; the sex of all of these individuals matched the molecular sex generated using this protocol.

*Calculation of growth rates and ages*

The von Bertalanffy growth function relates body size at recapture (SVL2) to previous size (SVL1) and the time spent growing between captures (Fabens 1965). A lizard’s age was estimated from the linear relationship between growth rate and initial body size, using size at birth (140mm SVL for both sexes) as the constant of integration (Van Devender 1978). We obtained the following age estimation equations:

males: ((1/-0.0033)*(LN(2.1105+(-0.0033*SVL))))+152

females: ((1/-0.0039)* (LN(2.1047+(-0.0039*SVL))))+112

These equations were used to estimate the age of each individual at the time of its first capture. We also reviewed published literature on ages at maturity in varanid lizards to compare our data to those available on other taxa.

**Results**

****Figure S1.** Mass relative to Snout-Vent Length (SVL) in male and female Yellow-Spotted Monitors (*Varanus panoptes*)

**Supplementary tables and figures**

**Table S1.** Data on growth rates (mass gains) in male and female *Varanus panoptes* at our field site, measured across certain periods of the activity cycle.

| **Animal ID** | **Sex** | **Age at first capture days** | **Last known age** | **Days since last measurement** | **Mass 1** | **Mass 2** | **Mass gain**  **(gm)** | **Period of growth** |
| --- | --- | --- | --- | --- | --- | --- | --- | --- |
| 2 | F | 263 | 408 | 103 | 780 | 1450 | 670 | Dec-Mar |
| 7 | F | 389 | 504 | 104 | 810 | 1340 | 530 | Dec-Mar |
| 9 | F | 292 | 401 | 102 | 620 | 840 | 220 | Dec-Mar |
| 11 | F | 503 | 860 | 99 | 850 | 1630 | 780 | Dec-Mar |
| 6 | F | 179 | 239 | 60 | 595 | 755 | 160 | Jan-Mar |
| 10 | F | 389 | 561 | 59 | 1280 | 1240 | -40 | Jan-Mar |
| 5 | F | 207 | 323 | 112 | 650 | 1015 | 365 | Jan-May |
| 8 | F | 207 | 329 | 116 | 835 | 1205 | 370 | Jan-May |
| 4 | F | 389 | 504 | 34 | 1330 | 1390 | 60 | Feb-Mar |
| 3 | F | 193 | 271 | 74 | 765 | 1160 | 395 | Feb-May |
| 1 | F | 222 | 291 | 65 | 1055 | 990 | -65 | Mar-May |
| 11 | F | 612 | 969 | 117 | 1630 | 1450 | -180 | Mar-July |
| 17 | M | 576 | 936 | 216 | 1860 | 3840 | 1980 | Dec-July |
| 22 | M | 278 | 651 | 110 | 1300 | 2700 | 1400 | Nov-Mar |
| 26 | M | 182 | 333 | 118 | 480 | 1370 | 890 | Nov-Mar |
| 18 | M | 126 | 306 | 152 | 410 | 1690 | 1280 | Nov-May |
| 13 | M | 505 | 873 | 106 | 2460 | 3840 | 1380 | Dec-Mar |
| 14 | M | 521 | 996 | 99 | 1630 | 3990 | 2360 | Dec-Mar |
| 15 | M | 597 | 997 | 106 | 1690 | 3690 | 2000 | Dec-Mar |
| 19 | M | 286 | 638 | 99 | 1460 | 1660 | 200 | Dec-Mar |
| 27 | M | 257 | 625 | 107 | 910 | 1680 | 770 | Dec-Mar |
| 12 | M | 557 | 1044 | 218 | 1980 | 3740 | 1760 | Dec-May |
| 21 | M | 376 | 597 | 149 | 1410 | 2440 | 1030 | Dec-Apr |
| 16 | M | 1138 | 1306 | 68 | 3920 | 4340 | 420 | Jan-Mar |
| 23 | M | 460 | 565 | 50 | 1890 | 2300 | 410 | Jan-Mar |
| 28 | M | 538 | 946 | 65 | 2100 | 2190 | 90 | Jan-Mar |
| 20 | M | 447 | 932 | 118 | 2060 | 2840 | 780 | Jan-May |
| 25 | M | 155 | 271 | 116 | 575 | 1065 | 490 | Jan-May |
| 20 | M | 521 | 1006 | 295 | 3040 | 3170 | 130 | July-May |
| 13 | M | 700 | 1068 | 112 | 3840 | 3940 | 100 | Mar-July |
| 14 | M | 521 | 996 | 112 | 3990 | 3140 | -850 | Mar-July |
| 15 | M | 644 | 1044 | 110 | 3690 | 3240 | -450 | Mar-July |
| 22 | M | 557 | 930 | 119 | 2700 | 3840 | 1140 | Mar-July |
| 27 | M | 294 | 662 | 111 | 1680 | 2040 | 360 | Mar-July |
| 28 | M | 557 | 965 | 115 | 2190 | 2940 | 750 | Mar-July |
| 29 | M | 264 | 335 | 67 | 1310 | 1595 | 285 | Mar-May |
| 20 | M | 489 | 974 | 69 | 2840 | 3040 | 200 | May-July |
| 24 | M | 489 | 836 | 46 | 2040 | 2340 | 300 | May-July |
| 15 | M | 856 | 1256 | 184 | 3240 | 3340 | 100 | July-Jan |

**Table S2.** Morphometric and age data for male and female *Varanus panoptes* in a large population on a tropical floodplain in northern Australia. Measurements provide means (plus standard error), minimum and maximum values of Snout to Vent Length (mm), Mass (gm), Body condition (Fulton’s K index) and growth rates (grams per day). Age at first capture was estimated from a Von Bertalanffy growth curve. As a proxy for longevity we added the additional days that an animal was tracked alive until they met a known fate or disappeared from the study (Female lizards n= 52; Male lizards n= 58).

| **Morphometric and age variables** | **Female *V. panoptes*** | | | **Male *V. panoptes*** | | |
| --- | --- | --- | --- | --- | --- | --- |
|  | *Mean (SE)* | *Min* | *Max* | *Mean (SE)* | *Min* | *Max* |
| Snout to Vent Length (SVL; mm) | 397 (7.7) | 140 | 510 | 501 (12.7) | 140 | 650 |
| Mass (gm) | 891 (42.6) | 35 | 1640 | 2105 (130.9) | 30 | 5050 |
| SVL (growth gm/day) | 0.3 (0.1) | 0.0 | 0.9 | 0.4 (0.1) | 0.0 | 2.2 |
| Mass (growth gm/day) | 2.9 (0.9) | -1.5 | 7.9 | 5.9 (1.2) | -7.6 | 23.8 |
| Body condition (Fulton’s K factor) | 1.39 (0.04) | 0.76 | 2.24 | 1.4 (0.04) | 0.80 | 2.40 |
| Age at first capture (days) | 272 (14) | 0 | 503 | 474 (39) | 0 | 1413 |
| Last known age (days) | 366 (20) | 108 | 860 | 642 (44) | 74 | 1497 |

**Table S3**. Predicted monthly prey intake by male and female yellow-spotted monitors, based on their average monthly energy requirements (provided in headings). The most common prey types of yellow-spotted monitors are included (as quantified for this population in Ward-Fear et al. 2020) with their associated mass and caloric content per gram of tissue. Each row represents the number of prey items required to support the total monthly energy budget of an average monitor lizard of that sex and size class (i.e., columns are not cumulative), at the 80% gross energy conversion efficiency (GEC) of varanid digestion. The daily rate of prey required for larger males in the population is given for foraging context.

| **Order** | **Common name** | **Prey energy content kj/gm** | **Average prey weight (gm)** | **Female (5491.4kj/ month)** | **Male (8170.2kj/ month)** | **Larger male (15100kj/ month)** | **Larger male forage per day** |
| --- | --- | --- | --- | --- | --- | --- | --- |
| Arachnidae | Wolf spider (*Lycosa laeta*) | 6.9 | 2 | 497 | 740 | 1368 | 46 |
| Chilopoda | Giant centipede (*Ethmostigmus rubripes*) | 6.8 | 50 | 20 | 30 | 56 | 2 |
| Blattodea | Cockroaches (*Cosmozosteria zonata*) | 7.9 | 8 | 109 | 162 | 299 | 10 |
| Gryllidea | Grasshoppers (*Gastrimargus musicus*) | 8.8 | 5 | 156 | 232 | 429 | 14 |
| Coleoptera | Cane beetle (*Lepidiota squamulata)* | 11.3 | 3 | 202 | 301 | 557 | 19 |
| Squamata | Gilberts lizard (*Lophognathus gilberti*) | 5.2 | 50 | 26 | 39 | 73 | 2 |
| Rodentia | Pale field rat (*Rattus tunneyi*) | 6.6 | 150 | 7 | 10 | 19 | 1 |
| Anura | Giant burrowing frog (*Cyclorana platycehpala*) | 2.87 | 40 | 60 | 89 | 164 | 5 |
| Passiriformes | Egg (wren/finch) | 29 | 1.5 | 158 | 235 | 434 | 14 |

**References**

Angilletta, M.J. and Sears, M.W. (2001). The metabolic cost of reproduction in an oviparous lizard. *Functional Ecology*, 14: 39-45.

Auffenberg, W. (1981). *The behavioral ecology of the Komodo monitor*. University Press of Florida.

Buffenstein, R. and Louw, G. (1982). Temperature effects on bioenergetics of growth, assimilaton efficiency and thyroid activity in juvenile varanid lizards. *Journal of Thermal Biology*, 7(4):197-200.

Christian, K.A., Corbett, L.K., Green, B. and Weavers, B.W., (1995). Seasonal activity and energetics of two species of varanid lizards in tropical Australia. *Oecologia*, 103(3):349-357.

Fabens, A.J. (1965). Properties and fitting of the Von Bertalanffy growth curve. *Growth*, (3):265-89.

Grayson, K.L., Cook, L.W., Todd, M.J., Pierce, D., Hopkins, W.A., Gatten, R.E. & Dorcas, M.E. (2005). Effects of prey type on specific dynamic action, growth, and mass conversion efficiencies in the horned frog, *Ceratophrys cranwelli*. *Comparative Biochemical Physiology*, 141:298-304.

Halverson, J. and Spelman, L.H. (2002). Sex determination. In: Murphy JB, Ciofi C, de la Panouse C, Walsh T (eds). *Biology and Conservation of Komodo Dragons*. Smithsonian Institute: Washington, DC, pp 165–177.

Peterson, C.C., Walton, B.M. and Bennett, A.F. (1999). Metabolic costs of growth in free-living Garter Snakes and the energy budgets of ectotherms. *Functional Ecology*, 13: 500-507.

Tinkle, D. W., and Hadley, N. F. (1975). Lizard Reproductive Effort: Caloric estimates and comments on its evolution. *Ecology*, 56(2): 427–434.

Van Devender, R.W. (1978). Growth ecology of a tropical lizard, *Basiliscus basiliscus*. *Ecology*, 59(5): 1031-1038.

Ward‐Fear, G., Shine, R. and Brown, G.P., 2020. Within‐population variation in dietary traits: implications for vulnerability and impact of imperilled keystone predators. *Ecosphere*, 11(10):e03136.

Wehrle, B.A. and German, D.P. (2023). Reptilian digestive efficiency: Past, present, and future. *Comparative Biochemical Physiology*, 277:111369.
